# What’s on the horizon?

**DOI:** 10.1093/immhor/vlaf001

**Published:** 2025-01-29

**Authors:** Bonnie N Dittel

**Affiliations:** Versiti Blood Research Institute, Milwaukee, WI 53226, United States

It is 2025, a quarter of the way through the 21st century, and we are on the cusp of an acceleration in the role of artificial intelligence in science. *ImmunoHorizons* was launched in 2017 and is now entering its ninth year. Nine-year-olds today are immersed in technology, and in their world at their fingertips they have instant access to information and technology that can solve problems for them. As the incoming Editor-and-Chief of *ImmunoHorizons*, it will be my responsibility to mature the journal and guide it into this new era. To accomplish this, a new slate of Section Editors will help steer the journal, and the new publishing agreement with Oxford University Press will bring new opportunities for growth. However, we cannot accomplish this alone. *ImmunoHorizons* is the American Association of Immunologists’ (AAI) open access journal that is uniquely positioned to provide timely content of interest to scientists interested in immunology. Given that the immune system plays a role in almost every organ, system, and disease in the body, advances in immunology are increasingly cross-disciplinary. My vision for the *ImmunoHorizons* of tomorrow is for it to be the go-to journal for the latest in immunology advances with content geared not only to “card-carrying” immunologists, but also to investigators whose research brings them to immunology. In other words, let’s bring immunology to the masses making immunology accessible to all.

This growth will take a continued commitment from the AAI and its members. A society journal is only as good as its members’ participation. I ask all AAI members, and anyone interested in immunology, to help us continue to build the journal. This can come in many forms, such as publishing your best work, reviewing, and promotion of the journal. My vision is that *ImmunoHorizons* is for everyone. It can be several firsts for young investigators: first publication, first reviewing experience, first commentary/editorial, or even first meeting report. Publishing in *ImmunoHorizons* is for all, as it serves the immunology community by funneling publication fees back to the AAI to support the many awards given to trainees and young investigators. If you see a role for yourself at *ImmunoHorizons*, please feel free to contact me with your ideas. It is an honor to be chosen to work with all of you over the next 5 years to continue to grow *ImmunoHorizons* into a sophisticated, tech savvy 14-year-old teenager that is on the verge of adulthood.

## Funding

None declared.

## Conflicts of interest

None declared.

